# Development and Analysis of Patient-Based Complete Conducting Airways Models

**DOI:** 10.1371/journal.pone.0144105

**Published:** 2015-12-11

**Authors:** Rafel Bordas, Christophe Lefevre, Bart Veeckmans, Joe Pitt-Francis, Catalin Fetita, Christopher E. Brightling, David Kay, Salman Siddiqui, Kelly S. Burrowes

**Affiliations:** 1 Computational Biology, Department of Computer Science, University of Oxford, Oxford, United Kingdom; 2 ARTEMIS Department, CNRS UMR 8145, Telecom SudParis, Institut Mines-Telecom, Paris, France; 3 Materialise NV, Leuven, Belgium; 4 Institute for Lung Health, Department of Infection, Immunity and Inflammation, University of Leicester, Leicester, United Kingdom; Technion - Israel Institute of Technology, ISRAEL

## Abstract

The analysis of high-resolution computed tomography (CT) images of the lung is dependent on inter-subject differences in airway geometry. The application of computational models in understanding the significance of these differences has previously been shown to be a useful tool in biomedical research. Studies using image-based geometries alone are limited to the analysis of the central airways, down to generation 6–10, as other airways are not visible on high-resolution CT. However, airways distal to this, often termed the small airways, are known to play a crucial role in common airway diseases such as asthma and chronic obstructive pulmonary disease (COPD). Other studies have incorporated an algorithmic approach to extrapolate CT segmented airways in order to obtain a complete conducting airway tree down to the level of the acinus. These models have typically been used for mechanistic studies, but also have the potential to be used in a patient-specific setting. In the current study, an image analysis and modelling pipeline was developed and applied to a number of healthy (*n* = 11) and asthmatic (*n* = 24) CT patient scans to produce complete patient-based airway models to the acinar level (mean terminal generation 15.8 ± 0.47). The resulting models are analysed in terms of morphometric properties and seen to be consistent with previous work. A number of global clinical lung function measures are compared to resistance predictions in the models to assess their suitability for use in a patient-specific setting. We show a significant difference (*p* < 0.01) in airways resistance at all tested flow rates in complete airway trees built using CT data from severe asthmatics (GINA 3–5) versus healthy subjects. Further, model predictions of airways resistance at all flow rates are shown to correlate with patient forced expiratory volume in one second (FEV1) (Spearman *ρ* = −0.65, *p* < 0.001) and, at low flow rates (0.00017 L/s), FEV1 over forced vital capacity (FEV1/FVC) (*ρ* = −0.58, *p* < 0.001). We conclude that the pipeline and anatomical models can be used directly in mechanistic modelling studies and can form the basis for future patient-based modelling studies.

## Introduction

Over the last decade there has been an increasing drive towards personalised and predictive healthcare through the development and application of computational models [1]. To date there have been relatively few studies using patient-specific models in multiple subjects, due predominantly to the effort involved in creating these models and in the ability to validate their patient-specific predictions. One example has been the application of computational models to understand the influence of patient-specific airway anatomy on lung function. Such models are increasingly being used within clinical trials and ultimately to support clinical decision making [2–4]. Typically these studies segment the airways from computed tomography (CT) images and generate computational models of multiple patients then compare the effect of airways geometry, after a given intervention, on simulated airflow. However, segmentation of airway geometries from CT images is limited to capturing only the larger airways, typically generations 6–10 on high resolution and generations 4–5 on low resolution CT. The conducting airways reach to generation 16, on average, and the full airway tree including the respiratory bronchioles includes approximately 23 generations of airway bifurcations [5]. This precludes the development of fully patient-specific airway geometric models based solely on CT images.

In order to study conditions where the small airways play an important role, including some forms of asthma and chronic obstructive pulmonary disease (COPD), models including the smaller airways (not visible via imaging) are required. These types of mathematical models of the complete bronchial tree have been used extensively to perform mechanistic studies of lung function through computational simulation. This work began with the morphometric measurements of Weibel [5], who developed a mostly symmetric model of airways structure that has been widely adopted by the lung community. Horsfield and co-workers produced the first asymmetric model of the airways, derived using information from resin casts [6], and further made detailed measurements of branching angles from the casts. Whilst the above studies provided detailed morphometric information they did not provide any regional structure to the models. A deterministic rule based algorithm was proposed by Kitaoka *et al*. [7] to generate a 3D airway tree into an idealised thoracic cavity. The method was based on a power law relationship between airway diameter and flow and produced visually appealing models. However, the resulting model was more asymmetric than the human lung and very sensitive to input parameters to the algorithm. Tawhai *et al*. [8] developed a space-filling algorithm to generate a 3D airway tree into separate host volumes defined by the lung lobes. The lobes were defined based on magnetic resonance images (MRI) of a male cadaver provided by the Visible Human Project. Airway diameters were randomly assigned based on Horsfield’s data [9]. Extensive validation of the morphometry of the resulting models was conducted showing good agreement with published data. This method was extended in [10] for use with CT data. Airway diameters were assigned using an optimal relationship based on the Strahler branching ratio of the tree and further morphometric validation was conducted. The algorithm also allows multiple starting branches to be used to grow into the host volume. Thus, it allows central airways extracted from the CT image to be merged into the complete algorithmically generated airway tree.

These mathematical models have been successfully used to understand mechanisms in a number of modelling studies [11–15]. However, they have rarely been used in a patient-specific setting in an analogous way to the central airways models derived directly from CT. Indeed, given that the generated airways are not truly patient-specific, but only patient-based, it is not clear whether using these models in a patient-specific setting is appropriate. Recent work, using *n* = 6 subjects, suggests that patient-based models of the type described here are required to accurately model FEV1 [16]. The study concluded that anatomical model creation is a time consuming process that would have to be streamlined to allow simulations on a large number of subjects.

Here we create a large number (n = 32) of patient-based complete conducting airway models generated from patient CT data. The models are analysed in terms of morphometric measures and to determine whether they are predictive of patient lung function through comparison of computational predictions with pulmonary function tests. To enable the study, a modelling pipeline was developed to streamline the process of model creation. The pipeline brings together software for airway and lobar segmentation, airway skeletonisation and generation of complete patient-based conducting airway trees to the level of the acinus. Finally, the utility of the models to form the basis of future patient-based or patient-specific studies in the context of larger modelling efforts [17] is discussed.

## Materials and Methods

### Patient Data

Acquisition of patient data has been described previously [18, 19]. 35 patients (n = 11 healthy, n = 24 asthmatic) were used for this study. Patient details and a summary of clinical characteristics are given in Table 1. Asthmatic patients were grouped by Global Initiative for Asthma (GINA) treatment step number [20]. Groups used were healthy, moderate asthmatic (GINA 1–2) and severe asthmatic (GINA 3–5). Patient spirometry measures (forced expiratory volume in one second (FEV1) and forced vital capacity (FVC)) were recorded post bronchodilator (BD).

**Table 1:** Clinical characteristics of study population.

	All	Healthy	GINA 1–2	GINA 3–5
Age (years)	55.69±14.07	54.27±14.53	65.71±8.52	52.47±14.32
Sex (M:F)	15:20	5:6	3:4	7:10
Body Mass Index	26.87±4.67	28.34±4.73	25.74±5.25	26.39±4.45
FEV1 Post BD	2.83±1.08	3.58±1.02	2.19±0.66*	2.61±1.02
FEV1/FVC Post BD	0.74±0.12	0.80±0.04	0.70±0.12	0.72±0.14
FEV1% Predicted	79.97±23.43	96.88±19.22	71.36±24.66	72.58±20.49*
FEV1/FVC% predicted	95.06±14.41	102.03±5.25	91.75±16.40	91.92±16.57

Groups were compared with a Kruskal-Wallis test with post-hoc pairwise comparison performed using a Mann-Witney *U*-test with Bonferroni correction. Post bronchodilator FEV1 and FEV1% predicted were seen to have statistically significant relationships between the three patient groups.

* *p* < 0.01 compared with healthy control.

#### CT scan acquisition

CT scans were acquired with a Siemens Sensation 16 scanner at 120kVp, 40 mAs, 1.5 pitch, 0.75 mm collimation and images reconstructed through a 512x512 matrix with a field of view targeted to image only pulmonary areas, 0.75 mm slice thickness, 0.5 mm slice interval. A B35f reconstruction algorithm was used for the CT data used in the current study. Inspiratory and expiratory volumetric CT scans of the thorax were performed with the subject in the supine position, arms held over their head and adequate breath-hold rehearsed prior to the scan. All subjects received bronchodilator therapy (2.5 mg nebulised Salbutamol no more than 1 hour prior to the scan). While both full inspiratory and expiratory scans were taken, only the inspiratory scans were used in this study. The study protocol was approved by the National Research Ethics Committee East Midlands Leicester (approval number 08/H0406/189), and all subjects gave their written informed consent.

#### Lung and Lobar Segmentation

In CT scans lungs show up as objects with lower density in relation to their surroundings. In brief, segmentation is obtained as follows: A global threshold is applied to define the shape of both lungs. Separation of the left and right lung is now required and proceeds in two steps. First a mask of the airways is subtracted from the combined lung field. Secondly, separation of the lung fields is achieved by applying a grey value based watershed. The final segmentation of the left and right lung field is obtained by applying classic watershed on the separated lungs.

Human lungs are separated into lobes by anatomical fissures that when complete are impermeable to air. They appear in CT images as bright surfaces on the darker background of the parenchyma. The detection of fissures is challenging, as they are thin and are often incomplete or blurred due to pathology, artifacts and the partial volume effect. In brief, the segmentations are obtained as follows. The fissure surface can be defined as the loci of points with high image intensity and positioned along the direction of greatest negative curvature [21]. A single-scale particle system was applied in order to sample all ridge surfaces in the CT images [22]. Three filtration stages were applied to filter out particles that do not belong to a lung fissure. In the first stage all particles were split into regions based on the distance and orientation, all small regions were discarded. Secondly anatomical prior knowledge was applied to leave only those particles that form a fissure-like surface. Finally a thin plate spline surface was fitted through the filtered regions of particles; regions that increased the bending energy of the surface significantly were not considered in the final set of particles. Thin plate spline surface built through the final set of particles was used to split the lungs into lobes. Results of the segmentation were assessed visually against the original CT scan to ensure accuracy.

### Airway Segmentation and Centreline Extraction

The airway segmentation technique has been described previously [23–25]. In brief, segmentation was performed in a multi-resolution iterative framework to maximise the reconstruction depth and minimise the “leakages” into the pulmonary parenchyma, where the parameters of the reconstruction scheme were automatically adapted to the CT acquisition protocol used. The approach exploits the topographical connectivity existing between the airway components in order to detect a 3D airway candidate subset, from which the airway tree is segmented by constrained region growing. After trachea detection, a flooding scheme is applied in the 3D CT dataset which provides a primary candidate set for the airways. An artifact cleaning procedure detects components of non-compliant geometry and selects as valid airway candidates the components connected to the trachea. The 3D reconstruction of distal airways is obtained from the selected candidates by means of constrained region growing combining several propagation schemes that are applied iteratively, until convergence [24].

Based on the obtained segmentation, a one dimensional model of the airway lumen geometry is built-up by means of its medial axis computation [25]. First, a disconnected point subset belonging to this axis is detected using a skeletonisation approach which adapts the Lantuéjoul formula [26] for an implementation based on the 3D discrete Euclidian distance function. An interconnection of these points is then performed using a minimum path search constrained by Euclidean and geodesic metrics, which guarantee a good axis centrality and a correct subdivision hierarchy. The medial axis is achieved by filtering out fake segments coming from lumen surface irregularities, and smoothing the final result [25]. The minimal lumen radius, given by the discrete Euclidian distance from the axis to the airway surface, is associated to each point on the axis. Whilst airway lumen cross-sectional area or hydraulic diameter would have been preferable to minimum radius, they could not be obtained automatically around bifurcation points. A large number of centreline segments are used to represent each airway and so longitudinal heterogeneity in airway radius is retained. Although, the airway segmentation method is automatic the results were assessed visually against the original CT scan for accuracy.

### Algorithmic Generation of Distal Airway Centrelines

An airway generation algorithm based on Tawhai *et al*. [10] was developed to allow generation of airway trees to the acinar level from the CT segmentations. The algorithm described in Tawhai *et al*. was chosen as a basis for the current study due to its extensive morphometric validation and its ability to have multiple starting branches within each lung lobe, allowing us to retain the maximum amount of information from the CT based airway geometries. The algorithm is volume filling and extrapolates the airway tree into the volume defined by the segmented lobar surface definitions. A number of modifications were made to the base algorithm.

1. The algorithm described here works directly with the segmented lobar triangular surface definition and central airways centrelines and so does not require the manual fitting of high-order meshes to image segmentations. A ray-casting technique was used to determine if a given point is inside or outside of each lobe.
2. The assignment of extrapolated lumen radii was based on the radius of the distal most parent branch from the central airways data, rather than assigned globally from the size of the trachea. This is described in detail in the next section.
3. An adaptive threshold on the distance between seed points and growth apices was found to be required to prevent a small number, typically 2–3 per subject, of spurious long airways being created in the last few generations. The threshold is given by
  $$T=\max(V_{b}-D_{l}*n,5\;\text{mm}),$$ (1)
  where *V*_*b*_ is the size of the diagonal of the bounding box of the lobe being generated into, *D*_*l*_ is the distance limit, typically *D*_*l*_ = *V*_*b*_/*N*, where *N* is the maximum number of generations, and *n* is the current generation number. The threshold used exploits the expected reduction in length observed between generations in morphometric measurements of the airway tree.

In brief, the generation process was as follows: A uniform grid of seed points was created within each segmented lobar surface definition. Each seed point roughly corresponds to a terminal bronchiole. Mean acinar volume in humans has been reported to be 187 mm^3^ [27]. Spacing of the seed point grid was set so that the mean volume around each seed point corresponded to this acinar volume. The distal ends of the segmented airway centrelines were used as starting points for the algorithm. While seed points remain, the algorithm proceeds as follows:

1. Each seed point is assigned to the closest distal branch within its lobe. To prevent spurious branches growing at the end of the generation, seed points are not assigned to a growth apex if they are further than the threshold distance *T* (see Eq (1)) from any apex. Points further than this threshold are discarded.
2. The centroid of the points assigned to each distal branch is calculated.
3. The plane defined by the centroid of the points and the nodes of the parent branch is used to split the points into two unequal sets.
4. The centroid of each of the new point sets is calculated.
5. For each set of points a new airway is generated starting at the distal end of the parent branch and extending 40% of the distance towards the centroid of the point set.
6. Each newly generated branch is checked to determine if it is terminal. Branches less than a set prescribed length (2 mm) are considered terminal. Branches whose point set contains only a single point are also considered terminal. For any terminal branch the closest seed point remaining is removed from the global set of seed points.
7. The process is repeated until no seed points remain.

### Assigning Diameters to the Generated Airways

The generated airways are idealised one-dimensional line segments and therefore must have lumen diameters assigned to them for use in simulation studies. Here a relationship based on Horsfield order, analogous to that described in [10] using Strahler order, is used to define the lumen diameter. The relationship defined by

$$\log D\left(x\right)=\left(x-N\right)\log\left(R_{d}H\right)+\log\left(D_{N}\right),$$ (2)

where *D* is the airway diameter, *x* is the current Horsfield order, *N* is the maximum Horsfield order, *D*_*N*_ is the maximum diameter and *R*_*d*_ *H* is the anti-log of the slope of the airway diameter plotted against Horsfield order and is set to 1.15.

Eq (2) extrapolates radii from a base airway with order, *N*, and diameter, *D*_*N*_. Two approaches for choosing this airway were tested: *tracheal* and *parent*. The tracheal approach assigns radii to the generated airways using the trachea as the base airway, as in [10]. The *parent* approach uses the parameters of CT based parent branch of each generated subtree. This has the effect of extrapolating abnormal airway luminal narrowing measured from CT-based airways, as seen in asthmatic patients, into the generated subtree. The possible advantages and shortcomings of this approach are addressed in the discussion.

### Morphometric Analysis

The following definitions were used in the analysis of the geometry of the airway tree data and models. The branching angle (*θ*) is the angle between parent and child branches. The rotation angle (*ϕ*) is the angle between the plane of the parent and its sibling branch and the plane made by the child branches. Branch diameter is denoted *D* and branch length is denoted *L*. The branch diameter for the branches derived from the CT data is the mean of the diameter along the length of the branch. For generated branches it is the diameter assigned using Eq (2). The minor child is defined to be the child branch with the smallest diameter, with the major branch being that with the largest diameter.

### Airflow Resistance Analysis

A zero-dimensional ventilation model using nonlinear resistance equations due to Pedley [28] and suitable for use at high Reynolds numbers was used to assess resistance to airflow in the airway trees. The airways were assumed to have a circular cross section, consistent with obtaining only the minimum radius of the airways from the CT scans. In the majority of cases this has shown to have a small impact on resistance predictions [29]. Longitudinal heterogeneity in airways radius was included in the resistance model. Resistance was calculated separately in both the CT derived central airways and the full conducting airway models. This allowed assessment of how the addition of the generated distal airway influenced total airway tree resistance and its correspondence to at the mouth measures of lung function.

For each airway the resistance is given by

$$R=\left\{\begin{array}{cc} ZR_{p} & \text{if}\;Z>1\\ R_{p} & \text{if}\;Z\leq 1, \end{array}\right.$$ (3)

where

$$Z=\frac{c}{4}\;{\left(Re\frac{d}{l}\right)}^{1/2},$$ (4)

*c* = 1.85, *l* and *d* are the length and diameter of the airway, *Re* is the Reynolds number and *R*_*p*_ is the Poiseuille resistance. The Reynolds number is given by

$$Re=\frac{4\rho |Q|}{\mu \pi d},$$ (5)

where *ρ* is the density of air at body temperature (1.15 Kg m^3^), where *μ* is the dynamic viscosity of air at body temperature (1.92e-5 Pa s) and *Q* is the flow rate through the airway. The Poiseuille resistance is given by

$$R_{p}=\frac{128\mu l}{\pi d^{4}}.$$ (6)

The Pedley based resistance model is in common use in for reduced dimensional lung ventilation models, see, for example, [12, 30, 31]. However, it is important to understand the assumptions and limitations of the model. Eq (3) was developed from studies of resistance in physical models of the bronchial tree that had at most four generations of airways. A constant area ratio between parent and child branches was used and each bifurcation had a fixed angle. The study only considered inspiration. Later work, however, has shown that Eq (3) can also be used to model expiration, albeit with an increase in the value of the *c* parameter [32]. Eq (3) only models the pressure drop due to viscous dissipation and does not consider the pressure drop due to kinetic energy changes. In healthy patients, pressure drops due to changes in kinetic energy are believed to be relatively small [33]. However, this may not be the case in the diseased case if the diameter of a parent airway is abnormally small relative to its children. Thus, the resistance calculated here can only be considered to be the resistance due to viscous dissipation rather than the total resistance of the airway tree. However, changes in frictional resistance are of most relevance for assessing diseased patients and it is the resistance due to viscous dissipation that is calculated via conventional body plethysmography [33].

Together with conservation of mass at each bifurcation, the resistance equations above give a non-linear problem that can be used to calculate flow in an airway tree. The problem was solved as follows: For an initial flow rate prescribed at the trachea, a linear system was formed to solve for pressure at each node and flow rate on each airway in the tree. Zero pressure boundary conditions were applied at the distal ends of the tree and an expiratory flow rate was prescribed at the entrance to the trachea. The linear system was solved using a direct solver and flow rates in the airway tree updated. Updates to flow rates were propagated to updates in all resistances using Eq 3. Fixed point iteration was applied to solve the full non-linear system until the *l*_∞_ norm of the relative residual was within a given tolerance. Full details of the numerical scheme are given in Section A in S1 File.

### Statistical Analysis

All statistical analysis was performed using R (www.R-project.org). All data are summarised as mean ± standard deviation. Intergroup comparisons between GINA classes were tested first with Kruskal-Wallis test, subsequently individual pairwise comparisons were calculated using the Mann-Whitney *U*-test with Bonferroni correction. Correlations between variables were examined using Spearman’s correlation coefficient. The method described in [34] was used to compare correlation coefficients (two-tailed). For all analysis, *p* < 0.01 was considered statistically significant.

### Software

Software to allow algorithmic generation of the distal airways and analysis of the resulting trees is available as part of the open-source Chaste (Cancer Heart and Soft Tissue Environment) package (www.cs.ox.ac.uk/chaste/). An add on package for Chaste including the software and models can be found at https://chaste.cs.ox.ac.uk/trac/wiki/PaperTutorials/AirwayGeneration2015. The airways were segmented using in house software described in [24, 25]. The lobes were segmented using the commercially available segmentation software Mimics (http://biomedical.materialise.com/mimics, Materialise NV, Technologielaan 15, 3001 Leuven, Belgium).

## Results

### Central Airways from CT

**Fig 1:**
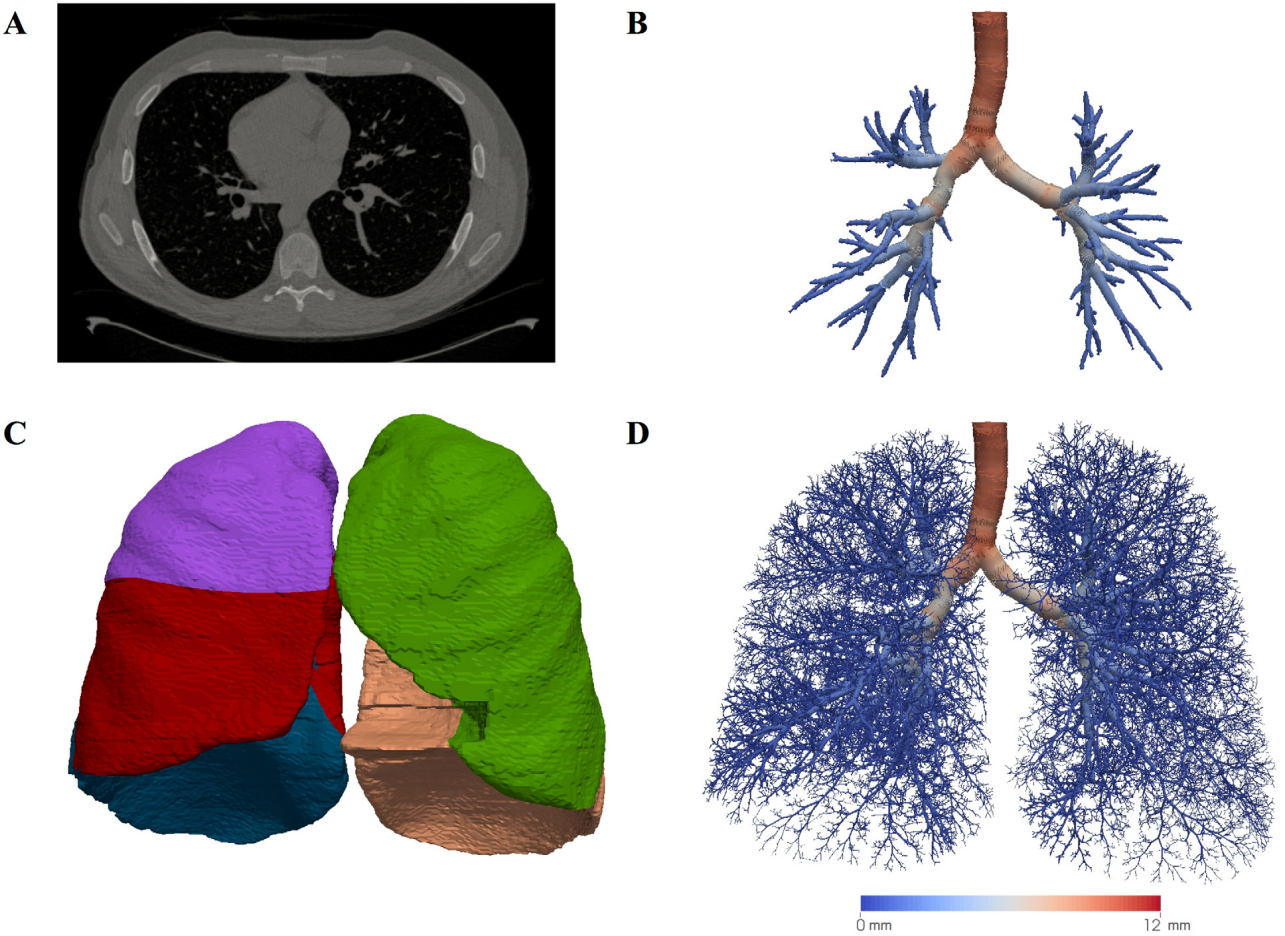
Model development. (a) An example cross-sectional slice through one of the CT images. (b) Central airways segmented from the same CT image. The segmentation consists of a centreline and minimum radius information, but is rendered as a series of tubes. The tubes are colour coded by the airway radius (see scale bar below (d)). (c) Lobar segmentations, a distinct colour is used for each lobe. (d) The complete conducting airway tree generated using the segmentations shown and the parent airway radius scheme. The tree is colour coded by airway radius (see scale bar).

Airway centrelines and minimum radii were successfully segmented from 34 out of 35 subjects (97% success rate). The mean generation number, across the subjects, of terminal segmented airways was 6.28 ± 0.73, the minimum was 3.97 ± 0.54 and the maximum 10.72 ± 1.69. An example segmentation is shown in Fig 1(b). A morphometric analysis of the segmented trees is given in Table 2. The trees are shown to be consistent with previous airway tree cast studies (Table A in S1 File). None of the morphometric parameters were shown to have statistically significant differences between the different patients Global Initiative for Asthma (GINA) classes. There was weak statistical evidence that *L*/*D* (*p* = 0.011) and *L*/*D*_*minor*_ (*p* = 0.013) varied between healthy patients and severe asthmatics (GINA 3–5), but this was below our threshold for significance. The standard deviation of *L*/*D* and related measures was seen to increase with the severity of asthma.

**Table 2:** Morphometric properties of segmented central airways.

	All	Healthy	GINA 1–2	GINA 3–5
*θ*	30.59±1.70	30.26±1.55	30.53±1.88	30.85±1.80
*θ* (*D*_*parent*_ > 4mm)	30.68±1.67	30.13±1.43	30.59±2.15	31.09±1.66
*θ* (4mm > *D*_*parent*_ > 3mm)	30.34±4.12	31.15±2.94	28.66±3.50	30.32±4.96
*θ* (3mm > *D*_*parent*_ > 2mm)	24.69±18.07	22.39±15.27	30.87±19.31	24.34±20.08
*θ* (2mm > *D*_*parent*_ > 1mm)	0.00±0.00	0.00±0.00	0.00±0.00	0.00±0.00
*ϕ*	90.00±0.00	90.00±0.00	90.00±0.00	90.00±0.00
*θ*_*minor*_	35.33±2.60	35.32±1.94	35.03±1.67	35.43±3.27
*θ*_*major*_	25.27±1.81	24.40±1.92	25.48±2.22	25.81±1.47
*L*/*D*	3.96±0.42	3.70±0.18	3.94±0.33	4.14±0.48^#^
*L*/*D*_*minor*_	4.45±0.51	4.15±0.31	4.41±0.36	4.67±0.57^#^
*L*/*D*_*major*_	3.46±0.43	3.25±0.22	3.40±0.40	3.62±0.49
*D*_*minor*_/*D*_*major*_	0.82±0.02	0.82±0.01	0.82±0.01	0.81±0.02
*D*/*D*_*parent*_	0.70±0.03	0.71±0.01	0.68±0.05	0.71±0.03
*D*_*minor*_/*D*_*parent*_	0.63±0.03	0.64±0.01	0.61±0.04	0.63±0.03
*D*_*major*_/*D*_*parent*_	0.78±0.03	0.79±0.02	0.75±0.05	0.79±0.04
*L*/*L*_*parent*_	1.57±0.22	1.52±0.16	1.57±0.04	1.60±0.28
*L*1/*L*2	0.48±0.04	0.50±0.03	0.48±0.06	0.47±0.04

Morphometric properties of the segmented central airways. All morphometric values are inline with values published from airway tree cast studies. Groups were compared with a Kruskal-Wallis test with post-hoc pairwise comparison performed using a Mann-Witney *U*-test with Bonferroni correction. There was weak statistical evidence that *L*/*D* and *L*/*D*_*minor*_ varied with patient GINA classification.

^#^ *p* < 0.02 compared with healthy control.

No other quantities were seen to vary.

### Lobes

The majority of the lobes were successfully segmented (33 of 35, 94% success rate). In one case the image quality was too poor to allow the segmentation process to be completed. In the second case the fissure between the right middle and lower lobes was ambiguous, resulting in inaccurate middle lobe segmentation. Fig 1(c) shows an example segmentation of the lobes from a single subject.

### Complete Generated Conducting Airway Trees

Complete patient-based airway trees were generated for all patients that had successful airway and lobar segmentations (32 of 35, 91% success rate for the whole pipeline). Fig 1(d) shows an example complete airway tree from a single patient using the parent radii scheme. The complete dataset is shown in Fig A in S1 File. Morphometric analysis of the complete (parent) airway trees is given in Table 3. All morphometric parameters were seen to be consistent with data from previous modelling studies and airway tree cast studies (Table A in S1 File). In common with the central airways trees, there were no statistically significant relationships between morphometric parameters and patient lung function.

**Table 3:** Morphometric properties of the generated airway trees.

	All	Healthy	GINA 1–2	GINA 3–5
*θ*	42.90±0.10	42.89±0.10	42.85±0.09	42.92±0.10
*θ* (*D*_*parent*_ > 4mm)	31.31±1.89	30.91±1.87	30.94±2.06	31.70±1.89
*θ* (4mm > *D*_*parent*_ > 3mm)	37.22±3.89	38.49±3.23	36.56±3.18	36.56±4.45
*θ* (3mm > *D*_*parent*_ > 2mm)	39.92±2.05	39.86±1.19	39.38±1.88	40.13±2.58
*θ* (2mm > *D*_*parent*_ > 1mm)	38.78±1.00	38.92±0.75	38.20±1.34	38.86±1.04
*ϕ*	90.00±0.00	90.00±0.00	90.00±0.00	90.00±0.00
*θ*_*minor*_	44.67±0.10	44.67±0.10	44.60±0.09	44.69±0.11
*θ*_*major*_	38.61±0.20	38.58±0.20	38.58±0.28	38.63±0.19
*L*/*D*	4.67±0.47	4.51±0.25	4.73±0.51	4.75±0.56
*L*/*D*_*minor*_	4.73±0.47	4.58±0.25	4.80±0.52	4.82±0.56
*L*/*D*_*major*_	4.51±0.45	4.37±0.24	4.56±0.50	4.60±0.55
*D*_*minor*_/*D*_*major*_	0.97±0.00	0.97±0.00	0.97±0.00	0.97±0.00
*D*/*D*_*parent*_	0.82±0.00	0.82±0.00	0.82±0.00	0.82±0.00
*D*_*minor*_/*D*_*parent*_	0.81±0.00	0.81±0.00	0.81±0.00	0.81±0.00
*D*_*major*_/*D*_*parent*_	0.85±0.00	0.85±0.00	0.85±0.00	0.85±0.00
*L*/*L*_*parent*_	0.89±0.00	0.89±0.00	0.89±0.00	0.89±0.00
*L*1/*L*2	0.68±0.00	0.68±0.00	0.68±0.00	0.68±0.00

Morphometric properties of the complete conducting airway trees. All morphometric values are inline with previous airway generation approaches and values published from airway tree cast studies. Groups were compared with a Kruskal-Wallis test. No quantities were seen to have a statistically significant relationship with patient GINA classification.

### Comparison of Airways Resistance with Patient Data

Airways resistance was calculated for all airway trees at low (0.17 *L*/*s*), moderate (0.83 *L*/*s*), and high (1.67 *L*/*s*) flow rates and using Poiseuille resistance for the central, complete (tracheal) and complete (parent) airway models. Correlations between the calculated resistances and patient spirometry measures were performed (Table 4). No statistically significant correlations were observed between the complete (tracheal) models and patient data.

**Table 4:** Correlations between model airways resistance and clinical measures.

	Resistance vs FEV1			Resistance vs FEV1/FVC		
Flow Rate (*L*/*s*)	Central	Complete (tracheal)	Complete (parent)	Central	Complete (tracheal)	Complete (parent)
Poiseuille	-0.56**	-0.10	-0.60**{0.49}	-0.44	0.08	-0.58**{0.075}
0.17	-0.52*	-0.21	-0.64**{0.05}	-0.31	0.026	-0.56*{0.066}
0.83	-0.53*	-0.30	-0.65**{0.01}	-0.32	-0.33	-0.42{0.075}
1.67	-0.53*	-0.31	-0.65**{0.01}	-0.32	-0.045	-0.42{0.075}

Spearman’s *ρ* correlation between model airways resistance and clinical spirometry measures. Airways resistance is calculated at several flow rates and for both the central and complete conducting airway models.

** indicates *p* < 0.001,

* indicates *p* < 0.01.

{} values indicate *p* value that the correlation is superior to the corresponding correlation for the central airways(two-tailed). Statistically signficant correlations were observed between resistance at all flow rates and patient FEV1, with the correlation being more marked for the complete conducting airway model. At low flow rates a statistically significant correlation was observed with resistance in the complete conducting models and patient FEV1/FVC.

**Fig 2:**
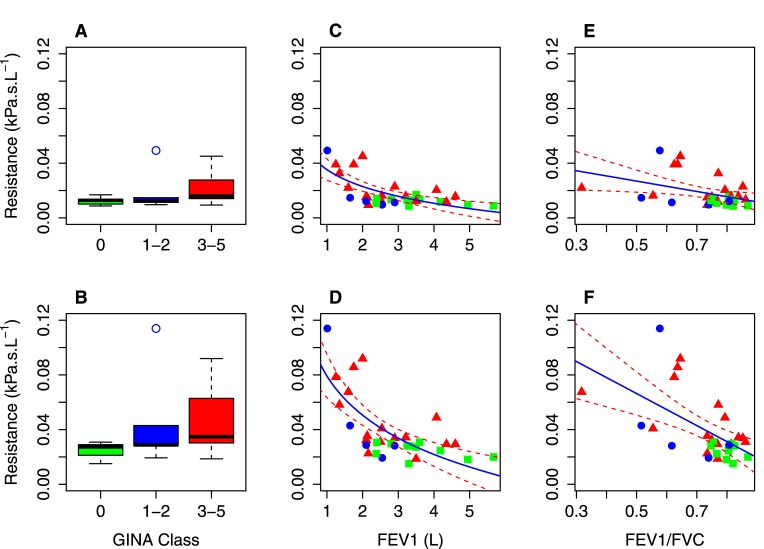
Model clinical data comparison. (Comparison between resistance at a flow rate of 0.17 L/s in the central airways model from CT data and the complete (parent) airways model with clinical data. Top row shows central airways, bottom row shows complete (parent) airways. Panels A & B show comparison of resistance with GINA Class, in both cases statistically significant (*p* < 0.001) differences were observed between resistances in healthy and severe (GINA 3–5) ashthmatics. Panels C & D show correlation between resistances and patient FEV1. In both cases moderate statistically significant correlations were observed (central: *ρ* = −0.52, *p* < 0.01, complete (parent): *ρ* = −0.64, *p* < 0.001). Panels E & F show correlation between resistances and FEV1/FVC. A mild statistically signifcant correlation was observed with airways resistance in the complete (parent) model: *ρ* = −0.56, *p* < 0.01). In all panels colour indicates patient GINA classification (Green: Healthy, Blue: GINA 1–2, Red: GINA 3–5). In panels C-F the solid line is a logarithmic regression (C-D) or linear regression (E-F) and the dashed lines are 95% confidence intervals for the regression. Regression coefficients were (C: Intercept (b) = -0.018, Slope (a) = 0.0035, D: b = 0.046, a = -0.038, E: b = 0.079, a = -0.041, F: b = 0.12, a = -0.12.

Moderate, statistically significant, correlations between patient FEV1 and resistance at all flow rates were observed in the central and complete (parent) models. There was statistical evidence that correlation with FEV1 was stronger in the complete conducting airway trees than in the imaging derived central airways alone (*ρ* = −0.65, *p* < 0.001 vs *ρ* = −0.53, *p* < 0.01 at 1.67 *L*/*s* flow rate), however this was just below our threshold for significance (*p* = 0.0102 at 1.67 *L*/*s* flow rate). Correlation was also seen to be slightly stronger at the high flow rate, although this was not statistically significant. Example correlation plots at the 0.17 *L*/*s* flow rate, are shown in Fig 2(c) and 2(d).

Moderate, statistically significant, correlation was observed between patient FEV1/FVC and resistance in the complete (parent) airway models at low flow rates (Table 4). This correlation was seen to decrease as the flow rate increased. No statistically significant correlations were observed between patient FEV1/FVC and resistance in the imaging derived central airways models. Example correlation plots at the 0.17 *L*/*s* flow rate, are shown in Fig 2(e) and 2(f).

A comparison of resistances at all flow rates in the airway models in the healthy, GINA 1–2 and GINA 3–5 groups using the Kruskal-Wallis was performed. No statistically significant differences between the groups were observed in the complete (tracheal) models. The test showed a statistically significant difference in resistance between the groups in both the central and complete (parent) models (*p* < 0.01). Further inter-group comparison showed a statistically significant (*p* < 0.001) change between the resistance in the severe asthmatics (GINA 3–5) and healthy patients. A small increase was observed in the mild asthmatics (GINA 1–2) over healthy in both cases, but this was not statistically significant. Example box plots at the 0.17 *L*/*s* flow rate, are shown in Fig 2(a) and 2(b).

**Fig 3:**
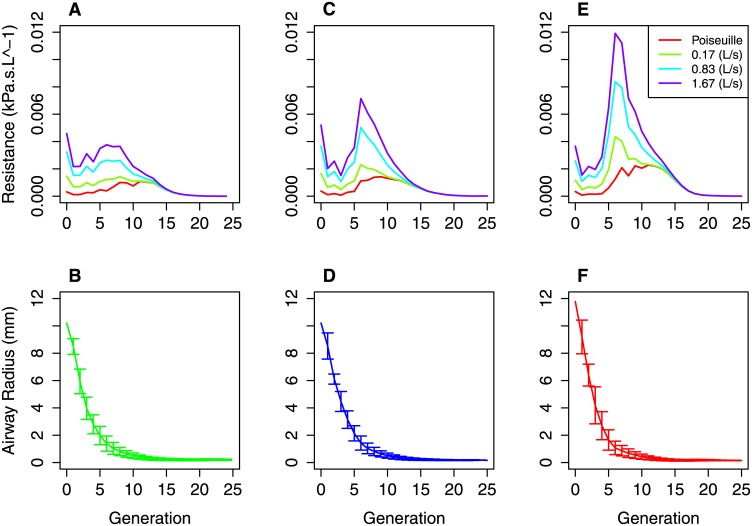
Representative flow resistance at different flow rates in three of the patient based models. Top row shows the contribution of each generation in the complete (parent) conducting airway models to the total airways resistance at the different tested flow rates. Three representative models are shown in the healthy (A), GINA 1–2 (C) and GINA 3–5 (F) groups. Bottom row shows the corresponding airway radii in each generation in each of the three representative models (Healthy (D), GINA1–2 (E), GINA 3–5 (F)). Error bars show standard deviation of the radii within a generation.

**Fig 4:**
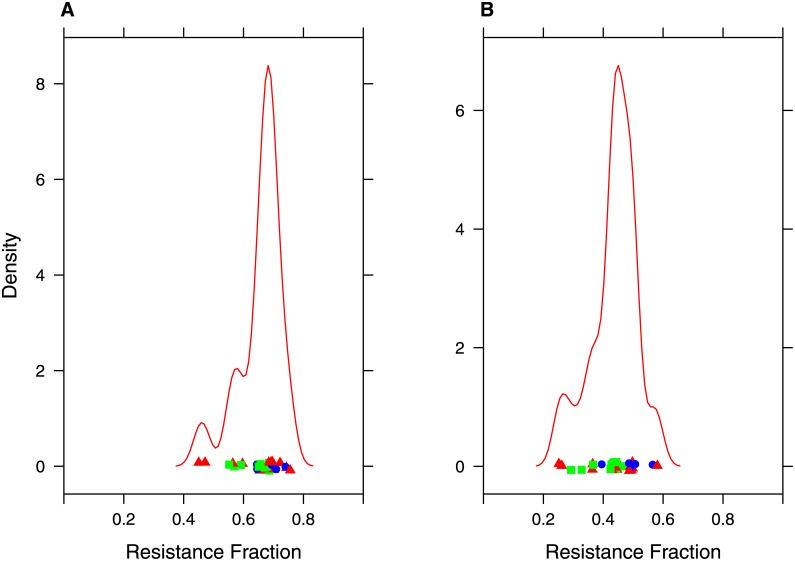
Flow resistance in imaging and generated portions of the airway tree. Analysis of contribution of the central and generated airways to total airflow resistance in the airway trees. Panels A and B show a kernel density plot of the fraction of total airflow resistance in the complete conducting models contributed by the algorithmically generated airways using Poiseuille resistance (A) and resistance at the high flow rate (1.67 L/s), colour indicates patient GINA classification (Green: Healthy, Blue: GINA 1–2, Red: GINA 3–5). A mean of 66% (Poiseuille resistance) to 44% (high flow rate) can be attributed to the generated airways. Similar plots for each of the individual groups are shown in Fig B in S1 File.

Fig 3 shows example distributions of resistance by airway generation in complete (parent) conducting airway tree models from the healthy, GINA 1–2 and GINA 3–5 groups at the different flow rates. The distributions are seen to be qualitatively similar to others in the literature based on idealised models of the airway tree [35]. As expected, the per generation resistance is generally higher in the moderate and severe groups than the healthy. The Figure also shows mean airway radii in each generation in the corresponding models. Whilst large changes in resistance are seen the differences in mean airway radii are much smaller. There is trend towards increased variance in radii in each generation, suggesting that the increased resistance is due to heterogeneous rather than homogenous airways constriction. Fig 4 shows the proportion of total tree resistance due to the extrapolated distal airways for each of the complete (parent) models at different flow rates. The mean proportion was 0.66 ± 0.07 for the Poiseuille flow model. This dropped to 0.44 ± 0.07 for the high flow rate. Thus, we see that the generated portion of the airways tree contributes significantly to the total flow resistance in our models, even at higher flow rates. A breakdown of the proportion in each of the patient subgroups is shown in Fig B in S1 File.

## Discussion

Computational fluid dynamics models with a high level of anatomical detail have been used to assess patient lung function from CT data. However, due to the limitations of CT and the computational difficulty of these simulations, such studies are typically limited to the first 6–10 generations. In contrast, complete conducting bronchial airway tree models (to generation ∼16) that are part based on CT data and part based on computational algorithm have previously been employed to study mechanisms underlying a variety of phenomena in the lungs. These include predictions of airways mechanics, gas mixing and pressure drops due to tidal breathing [11–15]. Complete conducting airway tree models have occasionally been used in a patient-specific setting in conjunction with functional imaging data such as positron emission tomography (PET) and hyperpolarised gas MRI [36, 37]. In neither case was the complete airways tree developed from matched CT data from the same patients as the functional images. As both PET and hyperpolarised gas MR largely quantify ventilation in the most distal regions of the lungs, the resulting personalised models are likely to be most accurate in the distal airways whilst lacking patient-specific data in the central airways. This is in comparison to a complete model generated from patient CT, as described here, which is most accurate in the central airways. In this study we have determined whether complete generated airway trees part based on CT data are advantageous for use in a patient-specific setting even without further use of functional imaging data.

To accomplish this goal an image processing and airways generation pipeline was constructed. The pipeline was used to segment high resolution CT and generate complete models for 32 asthmatic and healthy patients. The resulting models were analysed to verify that their morphometric properties are consistent with those available from airway tree cast studies and to determine how the models predicted airways resistance compares to patient spirometry measures.

### Airways and lobes from CT data

All central airways centrelines and the majority of lobes were successfully segmented from the CT images. The central airways segmentations were seen to be consistent with previous airways studies using a number of morphometric measures. There was weak statistical evidence that the relationship between airway length and diameter (*L*/*D*) varied between the patient groups. The lack of a strong relationship in this area may be due to the heterogeneity of asthma as a disease. Visual inspection of airways in the models shows both abnormally constricted and normal airways in the asthmatic patients. Thus, the difference in *L*/*D* between the groups may be reduced due to averaging. This is further supported by an observed increase in the standard deviation of *L*/*D* in the asthmatic groups.

Interestingly, the resistance of central airways centreline models was seen to have a moderate, statistically significant, correlation with patient FEV1 score at all tested flow rates. This is to be expected, as airways resistance depends on airway diameter and these are seen to reduce in asthma [19, 38]. However, our analysis shows that whilst there is a trend towards mean reduction in airway diameter between the asthmatic groups that trend is not statistically significant. Instead, our results suggest that changes in airways resistance between the groups are caused by heterogeneous airways constriction that is not readily seen in averaged data. These results are also in agreement with previous 3D CFD modelling studies, which observed slightly stronger correlation between airways resistance and FEV1 (Pearson’s *r* = −0.73, *p* = 0.003) [39]. Whilst the relationship between our calculated resistance and FEV1 is similar to previous work it must be noted that the correlation is somewhat weaker. There are three possible factors that may explain this. The first is that the image segmentations in [39] were cleaned manually, rather than automatically as in our case. The second is our central airways models only make use of the minimal lumen radius rather than true airways cross-section or hydraulic diameter. The use of minimal radius results in resistance predictions that are biased to be greater than otherwise. The third is that we are using a relatively simple model of airways resistance rather than a three-dimensional computational fluid dynamics model. However, the relationship between our model-predicted airway resistance and patient data is still present and the possible loss in accuracy a fair trade-off when using a with minimal manual intervention required.

We note that the CT scans used to build the models were taken post-bronchodilator and so the resulting models are not expected to include the effects of acute bronchoconstriction. The patient spirometry measures were also taken post-bronchodilator. Thus, comparison of airways resistance in the models to patient spirometry measures is consistent. However, in the absence of bronchodilators the patients may experience elevated bronchoconstriction and so reduced airway diameters. This effect may result in an increased GINA score in certain patients that cannot be directly detected in the CT derived models. Despite this, we still observed a statistically significant difference in airways resistance in the different patient groups.

### Complete conducting airway tree models

Complete airway models were successfully generated for all patients with airway and lobar segmentations (32 of 35). The complete models were consistent with both previous airways models and airway tree cast studies using a number of morphometric measures. Despite this, none of the morphometric measures were seen to have a statistically significant relationship with patient asthmatic status or at the mouth spirometry measures.

No correlation was found between resistance in the complete (tracheal) airway models and patient data. Resistance of complete (parent) airways models was seen to have a moderate, statistically significant, correlation with patient FEV1 score at all tested flow rates. A further significant correlation was observed with patient FEV1/FVC at all tested flow rates. Interestingly, correlations with spirometry data were better compared to those for just the central airways models, particularly correlation with FEV1/FVC. It is worth examining reasons why this might be the case.

One possible explanation is that our approach to assigning radii in the generated airway trees is dependent on the radius of the terminal segmented airways. There are two possible issues with this approach. The first is that normal airways may be visible on the CT whose child airways, not visible on CT, may be asthmatically constricted. In this case the model will underestimate the proportion of airways resistance in this part of the airway tree. The second issue is that abnormally constricted airways may be visible on the CT image whose child airways, again not visible on CT, are not abnormally constricted. In this case the resistance in the complete (parent) model will disproportionately reflect any luminal narrowing of the terminal airways visible on the CT. Clearly this approach cannot yield an anatomically exact match with the patients distal airways. However, extrapolating airway radii from the distal ends of the CT data maximises the impact of abnormal constriction seen in the CT data on the resulting models and our results suggest that doing so improves the correlation between resistance in the model and patient spirometry data.

Another possible explanation is that the complete (parent) conducting airway models are created by generating the airway tree into segmentations of the lobes. Thus, many properties of the complete airway tree models, including the level of asymmetry and the number of terminal bronchioles, are dependent on the size and shape of the patient’s lobes. This explanation is further supported by the comparative improvement in correlation with FEV1/FVC, which is heavily influenced by the size of the patients lungs, over correlation with FEV1 alone.

Analysis of the resistance of the complete (parent) airways models shows that, dependent on flow rate, between 44% and 66% of the total resistance is due to the generated portion of the tree. Thus, the generated portion of the airway tree adds significantly to the overall resistance in the model. Despite such a high proportion of the total airways resistance being from the generated portion of the airways tree we retain and indeed improve on correlations between total tree resistance and clinical data.

The airways resistance calculated here is only one metric of model validity and relevance. A number of three-dimensional models have been used to predict further aspects of lung function and their response to bronchoconstriction, such as regional ventilation distribution [12, 40, 41], FEV1 [16], the frequency dependence of ventilation [42–44]. Simulating these functional measures using, as a basis, the anatomical models described here would provide a greater understanding of the anatomical models’ validity as patient based models. Further, we believe that the cohort of models and model development pipeline described here, used in conjunction with previously developed functional models, has the long term potential to improve our understanding of patient lung function.

### Close

In this work we bring together existing methodologies to create an efficient modelling pipeline, the novelty in this study is the application of these techniques to a large number of well characterised patients and the comparison of modelling predictions to patient physiological data. We demonstrate that it is feasible to create a large number of patient-based models of the complete conducting airway tree from high-resolution CT data. We have shown that the models are morphometrically consistent with both previous modelling studies and with airway tree cast studies. We have shown that the predicted resistance to airflow in the complete (parent) airway tree models correlates with at the mouth spirometry measures and patient disease classification. This was not necessarily to be expected, as it was unknown if the addition of the algorithmically generated portion of the airway tree would have a detrimental effect on the relationship between airways resistance and patient clinical measures. This effect was observed in the complete (tracheal) models, whose resistance did not correlate with patient data. The models can be used in both mechanistic studies and as the basis for future patient-based models of the airways. Whilst we have shown that our models are patient-specific in terms of global at the mouth measures we have not simulated airflow in the models to predict regional ventilation nor compared predicted ventilation to functional images such as PET and hyperpolarised gas MRI. As such we cannot conclude that a complete airways model derived solely from CT is a sufficient representation of patient airways state to predict regional ventilation. However, we have shown that there is significant predictive power of patient global lung function (spirometry) measures in the complete patient-based models and this may be advantageous when used as a basis for future patient-specific modelling work.

## Supporting Information

S1 File Supporting Information. Description of the method used to calculate resistance in the conducting airway models (**Section A**). The complete patient-based conducting airway models (**Fig A**). The proportion of total airways resistance due to the generated distal airways (**Fig B**). The morphometric properties of the segmented central airways and complete conducting airway models (**Table A**).
(PDF)
