# Supplementary material for: Development and Analysis of Patient-Based Complete Conducting Airways Models
Source: PLoS One. 2015 Dec 11;10(12):e0144105. doi: 10.1371/journal.pone.0144105 (PMC4684353; doi:10.1371/journal.pone.0144105)
Supplement: S1 File — Description of the method used to calculate resistance in the conducting airway models (Section A). The complete patient-based conducting airway models (Fig A). The proportion of total airways resistance due to the generated distal airways (Fig B). The morphometric properties of the segmented central airways and complete conducting airway models (Table A). (PDF) [file pone.0144105.s001.pdf]

Development and analysis of patient-based conducting airway models: supporting information

A. Calculating resistance in airway tree models

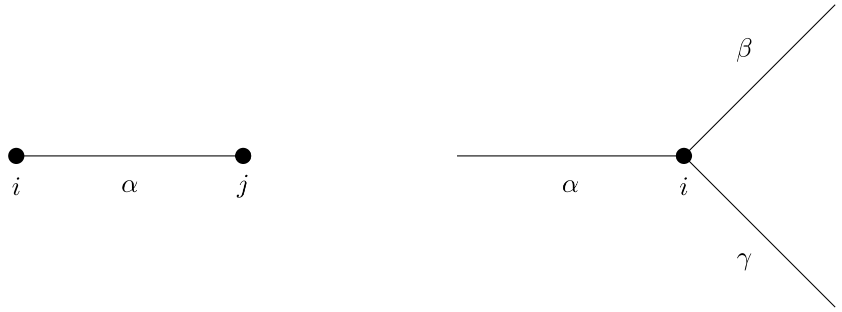

(a) Schematic of a single airway, α, connected to nodes, i, j.

(b) Schematic of a single bifurcation, i, connected to airways, α, β, γ.

Figure 1. Airway schematic diagrams

The conducting airway models are made up of a set of one-dimensional elements connected to nodes. The nodes represent either the entrance to the trachea, bifurcations or the terminal ends of the airway tree. A pressure, P_i is defined at each node, i, where $i = 1, \ldots, N_{nodes}$. Similarly, a flow rate, Q_α is defined on each element, α, where $\alpha = 1, \ldots, N_{eles}$.

For each airway, α, as shown in Figure 1a, the pressure-flow relationship is governed by

$$P_i - P_j = Q_\alpha R_\alpha, \tag{1}$$

where the resistance, R_α, is a function of flow rate given by

$$R(Q) = \begin{cases} Z(Q)R_p & \text{if } Z(Q) > 1 \\ R_p & \text{if } Z(Q) \leq 1, \end{cases} \tag{2}$$

where

$$Z(Q) = \frac{c}{4}\left(Re(Q)\frac{d}{l}\right)^{1/2}, \tag{3}$$

$c = 1.85$, l and d are the length and diameter of the airway, Re is the Reynolds number and R_p is the Poiseuille resistance. The Reynolds number is given by

$$Re(Q) = \frac{4\rho|Q|}{\mu\pi d}, \tag{4}$$

where ρ is the density of air at body temperature (1.15 Kg m^3), μ is the dynamic viscosity of air at body temperature (1.92e-5 Pa s). The Poiseuille resistance is given by

$$R_p = \frac{128\mu l}{\pi d^4}. \tag{5}$$

Pressure boundary conditions are applied at each terminal node such that $P_k = P_0$ for every terminal node k.

At each bifurcation, i, as show in Figure 1b, flow rate is conserved such that

$$Q_\alpha = Q_\beta + Q_\gamma. \tag{6}$$

A flow rate boundary condition is applied at the trachea, such that for the tracheal element, $Q_0 = Q_{in}$.

Equations 1 - 6 considered for each element and node form a non-linear system that can be solved to calculate pressures and flow rates on the airway tree. The non-linear system can be written in the form

$$\mathbf{A}(\mathbf{Q}) \begin{bmatrix} \mathbf{Q} \\ \mathbf{P} \end{bmatrix} = \mathbf{b}, \tag{7}$$

where $\mathbf{Q} = [Q_1, \ldots, Q_{N_{eles}}]^T$, $\mathbf{P} = [P_1, \ldots, P_{N_{nodes}}]^T$, $\mathbf{A}$ is a block matrix of the form

$$\begin{bmatrix} \mathbf{A}_{11}(\mathbf{Q}) & \mathbf{A}_{12} \\ \mathbf{A}_{21} & \mathbf{A}_{22} \end{bmatrix} \tag{8}$$

where $\mathbf{A}_{11} \in \mathbb{R}^{N_{eles} \times N_{eles}}$, $\mathbf{A}_{12} \in \mathbb{R}^{N_{eles} \times N_{nodes}}$, $\mathbf{A}_{21} \in \mathbb{R}^{N_{nodes} \times N_{eles}}$, $\mathbf{A}_{22} \in \mathbb{R}^{N_{nodes} \times N_{nodes}}$ and $\mathbf{b}$ is a block vector of the form

$$\begin{bmatrix} \mathbf{b}_1 \\ \mathbf{b}_2 \end{bmatrix}, \tag{9}$$

where $\mathbf{b}_1 \in \mathbb{R}^{N_{eles}}$ and $\mathbf{b}_2 \in \mathbb{R}^{N_{nodes}}$.

Entries in $\mathbf{A}_{11}$ and $\mathbf{A}_{12}$ are derived from equation (1) such that for each element α connected to nodes i and j, as shown in Figure 1a,

$$(\mathbf{A}_{11})_{\alpha,\alpha}(Q_\alpha) = -R_\alpha(Q_\alpha), \qquad (\mathbf{A}_{12})_{\alpha,i} = 1, \qquad (\mathbf{A}_{12})_{\alpha,j} = -1. \tag{10}$$

Entries in $\mathbf{A}_{21}$ are derived from equation (6) such that for each bifurcation node, i, connected to elements α, β, γ, as shown in Figure 1b,

$$(\mathbf{A}_{21})_{i,\alpha} = 1, \qquad (\mathbf{A}_{21})_{i,\beta} = -1, \qquad (\mathbf{A}_{21})_{i,\gamma} = -1. \tag{11}$$

The flow rate boundary condition at the tracheal element, 0, gives

$$(\mathbf{A}_{21})_{0,0} = 1, \qquad (\mathbf{b}_1)_0 = Q_{in}. \tag{12}$$

Pressure boundary conditions at each terminal node, k, gives

$$(\mathbf{A}_{22})_{k,k} = 1, \qquad (\mathbf{b}_2)_k = P_0. \tag{13}$$

All other matrix and vector entries are zero.

The full non-linear system can then be solved using a fixed point iteration of the form

Algorithm 1 Fixed point iteration to solve non-linear pressure and flow rate problem

$k = 0$
$\mathbf{Q}^0 = \mathbf{0}$
$\mathbf{P}^0 = \mathbf{0}$
repeat
  $k = k + 1$
  Solve $\mathbf{A}(\mathbf{Q}^{k-1}) \begin{bmatrix} \mathbf{Q}^k \\ \mathbf{P}^k \end{bmatrix} = \mathbf{b}$
until $\frac{||[\mathbf{Q}^k \ \mathbf{P}^k]^T - [\mathbf{Q}^{k-1} \ \mathbf{P}^{k-1}]^T||_\infty}{||[\mathbf{Q}^k \ \mathbf{P}^k]^T||_\infty} < TOL$

where TOL is the tolerance the solution is required to and a direct solver is applied to solve the linear system [1].

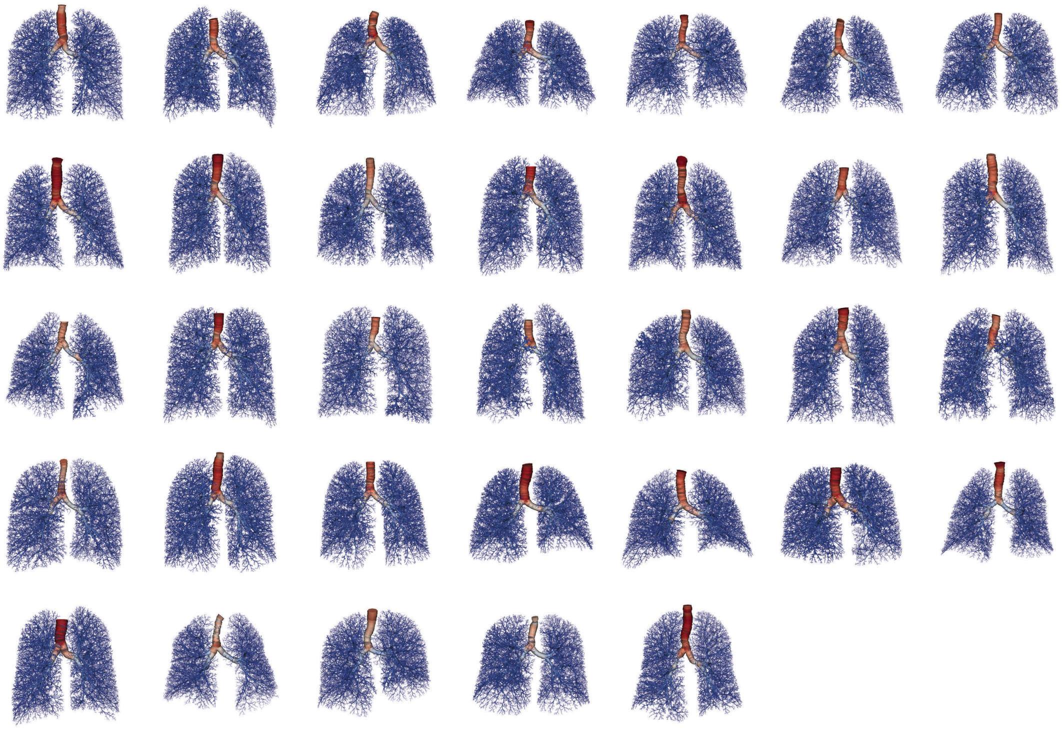

Figure A. Complete patient-based conducting airway models. Each image shows a patient-based model of the complete conducting airways. Each model is created using a CT scan of a unique patient and reflects that patientś airway structure.

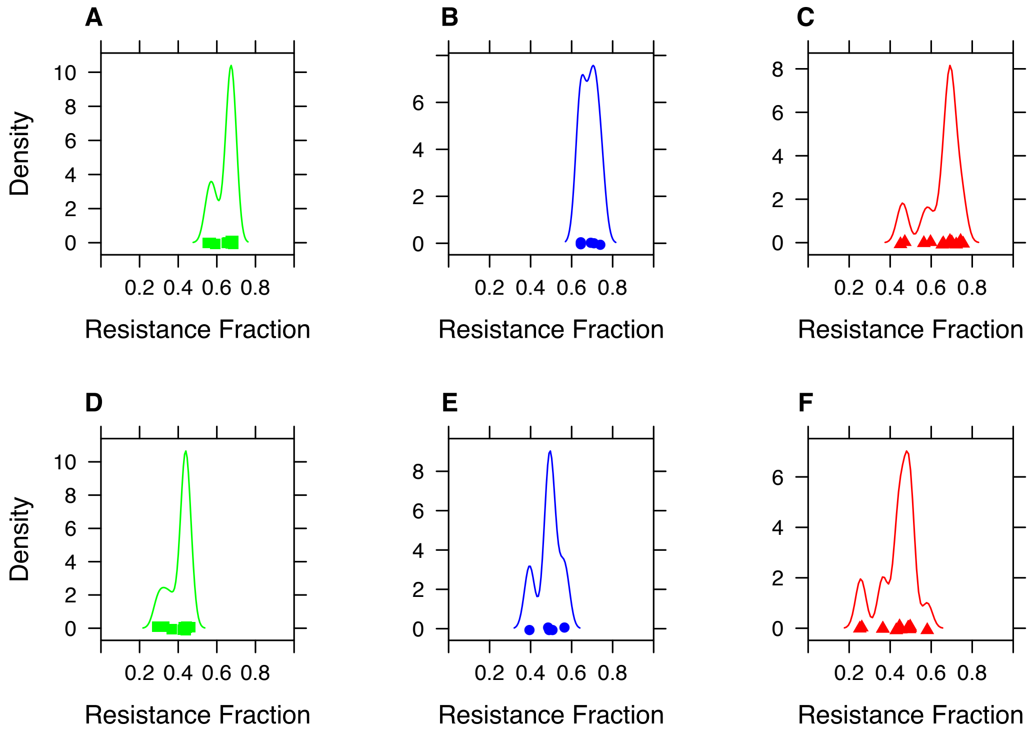

Figure B. Proportion of total airways resistance due to the generated distal airways. Panels A-C show the proportion of resistance in the Poiseuille flow model in Healthy (A), GINA 1-2 (B) and GINA 3-5(C) patients. Panels D-F show the proportion of resistance at the 1.67 L/a flow rate in Healthy (D), GINA 1-2 (E) and GINA 3-5(f) patients.

	CT Central Airways				Complete Conducting Airways				Published Measures	
	All	Healthy	GINA 1-2	GINA 3-5	All	Healthy	GINA 1-2	GINA 3-5	Experimental	Model
θ	30.59±1.70	30.26±1.55	30.53±1.88	30.85±1.80	42.90±0.10	42.89±0.10	42.85±0.09	42.92±0.10	37[2];39,43[3]	50.31[4]
θ (D_p>4mm)	30.68±1.67	30.13±1.43	30.59±2.15	31.09±1.66	31.31±1.89	30.91±1.87	30.94±2.06	31.70±1.89	32[5]	33.71[4]
θ (4mm>D_p>3mm)	30.34±4.12	31.15±2.94	28.66±3.50	30.32±4.96	37.22±3.89	38.49±3.23	36.56±3.18	36.56±4.4532	30[5]	36.72[4]
θ (3mm>D_p>2mm)	24.69±18.07	22.39±15.27	30.87±19.31	24.34±20.08	39.92±2.05	39.86±1.19	39.38±1.88	40.13±2.58	36[5]	45.19[4]
θ (2mm>D_p>1mm)					38.78±1.00	38.92±0.75	38.20±1.34	38.86±1.04	43[5]	53.34[4]
ϕ	90.00±0.00	90.00±0.00	90.00±0.00	90.00±0.00	90.00±0.00	90.00±0.00	90.00±0.00	90.00±0.00	79[3], 90[6]	89.99[4]; 90[7]
θ_{minor}	35.33±2.60	35.32±1.94	35.03±1.67	35.43±3.27	44.67±0.10	44.67±0.10	44.60±0.09	44.69±0.11		53.00[4]
θ_{major}	25.27±1.81	24.40±1.92	25.48±2.22	25.81±1.47	38.61±0.20	38.58±0.20	38.58±0.28	38.63±0.19		47.63[4]
L/D	3.96±0.42	3.70±0.18	3.94±0.33	4.14±0.48$^{\#}$	4.67±0.47	4.51±0.25	4.73±0.51	4.75±0.56	3.09,3.14[3];2.8-3.25[8]	2.92[4];3.0[7]
L/D_{minor}	4.45±0.51	4.15±0.31	4.41±0.36	4.67±0.57$^{\#}$	4.73±0.47	4.58±0.25	4.80±0.52	4.82±0.56	2.96[9]	2.88[4]
L/D_{major}	3.46±0.43	3.25±0.22	3.40±0.40	3.62±0.49	4.51±0.45	4.37±0.24	4.56±0.50	4.60±0.55	2.71[9]	2.96[4]
D_{minor}/D_{major}	0.82±0.02	0.82±0.01	0.82±0.01	0.81±0.02	0.97±0.00	0.97±0.00	0.97±0.00	0.97±0.00	0.82,0.74[3];0.86[8, 10]	0.81[4]
D/D_p	0.70±0.03	0.71±0.01	0.68±0.05	0.71±0.03	0.82±0.00	0.82±0.00	0.82±0.00	0.82±0.00	0.83,0.78[3];0.79[8]	0.79[4]
D_{minor}/D_p	0.63±0.03	0.64±0.01	0.61±0.04	0.63±0.03	0.81±0.00	0.81±0.00	0.81±0.00	0.81±0.00		0.69[4]
D_{major}/D_p	0.78±0.03	0.79±0.02	0.75±0.05	0.79±0.04	0.85±0.00	0.85±0.00	0.85±0.00	0.85±0.00	0.86[9]	0.88[4]
L/L_p	1.57±0.22	1.52±0.16	1.57±0.04	1.60±0.28	0.89±0.00	0.89±0.00	0.89±0.00	0.89±0.00	0.94[11]	0.81[4]
$L1/L2$	0.48±0.04	0.50±0.03	0.48±0.06	0.47±0.04	0.68±0.00	0.68±0.00	0.68±0.00	0.68±0.00	0.58[3];0.62[8, 10]	0.68[4]

Table A. Morphometric properties of the segmented central airways and complete conducting airway models Comparisons are shown with similar measures that have been published in the literature. These are divided into experimental measures (such as those obtained via histology and airway tree casts) and measures extracted from other lung models.

References

[1] Davis TA, Palamadai Natarajan E (2010) ACM Trans. Math. Softw., New York, NY, USA: ACM, volume 37, chapter Algorithm 907: KLU, A Direct Sparse Solver for Circuit Simulation Problems. pp. 36:1–36:17.

[2] Horsfield K, Cumming G (1967) Angles of branching and diameters of branches in the human bronchial tree. Bulletin of Mathematical Biology 29: 245–259.

[3] Sauret V, Halson PM, Brown IW, Fleming JS, Bailey AG (2002) Study of the three-dimensional geometry of the central conducting airways in man using computed tomographic (CT) images. Journal of Anatomy 200: 123–134.

[4] Tawhai MH, Hunter P, Tschirren J, Reinhardt J, McLennan G, et al. (2004) CT-based geometry analysis and finite element models of the human and ovine bronchial tree. Journal of Applied Physiology 97: 2310–2321.

[5] Horsfield K, Dart G, Olson DE, Filley GF, Cumming G (1971) Models of the human bronchial tree. Journal of Applied Physiology 31: 207–217.

[6] Horsfield K (1985) gas mixing and distribution in the lung, Dekker, volume 25 of *Lung biology in health and disease*, chapter Anatomical factors influencing gas mixing and distribution. pp. 23–61.

[7] Kitaoka H, Takaki R, Suki B (1999) A three-dimensional model of the human airway tree. Journal of Applied Physiology 87: 2207–2217.

[8] Weibel E (1963) Morphometry of the Human Lung. Berlin-Gottingen-Heidelberg: Springer Verlag.

[9] Phillips CG, Kaye SR (1995) Diameter-based analysis of the branching geometry of four mammalian bronchial trees. Respiration Physiology 102: 303–316.

[10] Phalen RF, Yeh HC, Schum GM, Raabe OG (1978) The Anatomical Record, Wiley Subscription Services, Inc., A Wiley Company, volume 190, chapter Application of an idealized model to morphometry of the mammalian tracheobronchial tree. pp. 167–176.

[11] Krause E, Bandt C, Schulz A, Schulz H (1995) Fractal exponents for the upper airways of mammalian lungs. Computational Statistics & Data Analysis 20: 583–590.
